# Evaluation of Folic Acid-Containing Mouthrinse and Chlorhexidine Mouthrinse as an Adjunct to Scaling and Root Planing in Patients With Periodontal Disease

**DOI:** 10.7759/cureus.64853

**Published:** 2024-07-18

**Authors:** Aashik C. R., Rajapandian K., Gayathri K., Ravishankar P. L., Kalaivani V., Sunanda Rao K.

**Affiliations:** 1 Periodontology, Sri Ramaswamy Memorial (SRM) Kattankulathur Dental College and Hospital, Chennai, IND

**Keywords:** scaling and root planing, mouthrinse, folic acid, chronic periodontitis, chlorhexidine

## Abstract

Background: Periodontal disease is a host-mediated inflammation caused due to microbial challenge. Hence, mechanisms involving the control of host-associated mediators can be a potential target. The conventional nonsurgical periodontal treatment modality includes scaling and root planing (SRP), which is often combined with adjunctive chemical plaque control agents for effective disease control. Chlorhexidine (CHX) is the most common chemical plaque control agent used. Recent research is now being focused on exploring other medicinal substitutes that may benefit control of inflammation and tissue healing. Folic acid is an important nutrient that increases the ability of oral epithelial cells to resist local irritants and inflammation if supplemented either systemically or locally.

Aim: The current study aimed to evaluate the effect of folic acid and CHX mouthwash as an adjunct to scaling and root planing for treating patients with chronic periodontitis.

Methodology: In this study, 30 patients with chronic periodontitis were included and assigned to either of the two groups: Group A (receiving folic acid-containing mouthrinse) and Group B (receiving CHX mouthrinse). Periodontal measurements, including plaque index, probing pocket depth, gingival index, and healing index, were evaluated at baseline and again four weeks after scaling and root planing.

Results: Significant reduction was detected in all clinical parameters (plaque index, gingival index, probing pocket depth, healing index) for both groups (p<0.05) when evaluated from baseline to four weeks.

Conclusion: Both mouthrinses were effective when used as an adjunct to scaling and root planing in the treatment of periodontitis. Hence, folic acid-containing mouthrinse can be used in patients with chronic periodontitis.

## Introduction

Periodontitis is a persistent and multifactorial inflammatory disease that impacts the tissues supporting the teeth mediated by host-microbial interaction in an environment conducive to it. It affects a substantial section of the population with a more than 50-97% prevalence in India. It manifests as erythematous gingiva with gingival bleeding, periodontal pockets, and gingival recession, all of which contribute to alveolar bone loss and tooth loss [1].

Dental plaque is recognized as the primary factor in the initiation and progression of periodontal disease, making its control essential [2]. The two fundamental subcategories into which plaque control can be split are mechanical and chemical [3]. Scaling and root planing are steps in the mechanical debridement process that remove plaque and calculus from the tooth surface. For better treatment outcomes, chemical plaque control agents are frequently employed in conjunction with mechanical debridement. Routine oral plaque control agents are being used in the form of mouthwashes, gels, dentifrices, chewing gums, etc. [4].

The most commonly used chemical plaque control agent is chlorhexidine (CHX). The antibacterial action of CHX is dose-dependent, with bacteriostatic effects at concentrations between 0.02% and 0.06% percent and bactericidal effects at doses over 0.12% [5]. There is a concern that CHX mouthwash may negatively affect gingival fibroblasts, altering their morphology, viability, and function. These detrimental effects have been noted even at low concentrations, such as a 25% dilution of commercially available mouthwashes. In addition to this, periodontal disease exhibits alteration in the host's resistance that can modify the integrity of the dentogingival barrier and cell turnover. To overcome this, agents with improved tissue healing properties that can have beneficial effects on periodontal disease are now being explored [6,7].

Folic acid is required for the maturation and proliferation of cells. Nutrition Examination Survey data evaluated the relationship of folic acid inadequacy to periodontal disease severity and suggested that its supplementation reduced the disease severity. When folic acid deficiency was studied histologically, it showed the inability of epithelial cells to develop, impairment of the keratinization process, and susceptibility to ulceration and secondary infection [8]. This indicates that folic acid deficiency would also affect the gingival epithelium. Though CHX has predominant antimicrobial properties and is most commonly used as a plaque control agent in periodontitis patients, compounds like folic acid may be employed to promote healing and repair the dentogingival barrier, which could contribute to improved periodontal therapeutic outcomes. Previous studies have focused on the beneficial effects of folic acid on periodontal tissue when given as a systemic supplement [9,10]; however, the evaluation of folic acid-containing mouthrinse as an adjunct to non-surgical periodontal therapy is yet to be explored. Therefore, the purpose of the present study is to assess the effectiveness of commercially available folic acid mouthwash in acting as an adjuvant to scaling and root planing in patients with periodontitis.

## Materials and methods

Study population

The present study was carried out at the Department of Periodontology, Sri Ramaswamy Memorial (SRM) Kattankulathur Dental College and Hospital, Chennai, Tamil Nadu, India (approval no.: SRMIEC-ST0722-51). Before the study initiation, informed consent was obtained from each patient. A total of 30 participants were included and subdivided into groups A and B, respectively. Group A participants received folic acid-containing mouthwash PerioClear® (Nature’s Answer Products Ltd., New York, USA), whereas Group B received CHX mouthwash Hexidine® (ICPA Health Products Ltd., Mumbai, India) containing chlorhexidine gluconate (0.2% w/v).

The participants were allocated into groups using a simple lottery procedure, and the subjects received their assigned mouthrinses. Complete subject masking was made possible by using identical bottles for each mouthwash that was used. For four weeks, the subjects used 10 mL of mouthwash twice daily for one minute after brushing their teeth.

Inclusion criteria and exclusion criteria

Inclusion criteria: Patients between the ages of 18-50 years. Stage II and III, grade B periodontitis were diagnosed based on the criteria from the 2017 World Workshop on the Classification of Periodontal Diseases Consensus Report. Consequently, the study included patients with a probing depth (PD) of at least 6 mm and a clinical attachment loss (CAL) of at least 5 mm.

Exclusion criteria: Patients who have undergone periodontal treatment in the past six months, patients on antibiotic or immunosuppressant medication six months before the study, smokers and alcoholics, pregnant and lactating women, medically compromised patients, teeth with hopeless prognosis.

Assessment of the clinical parameters

**Figure 1:**
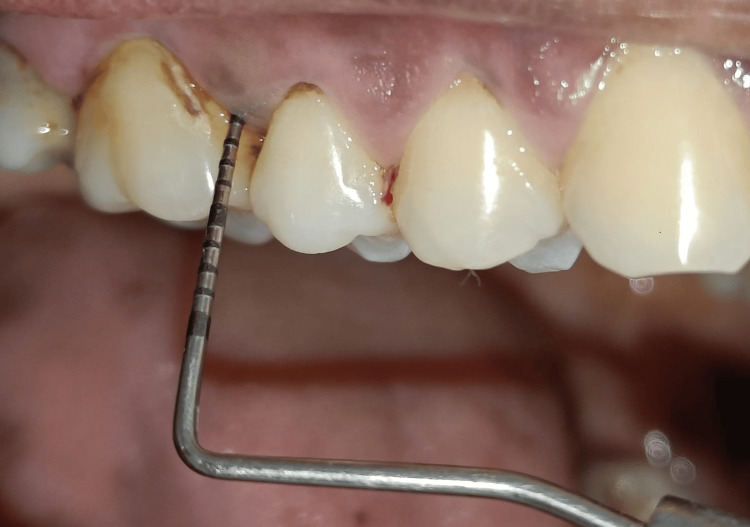
Preoperative periodontal examination

**Figure 2:**
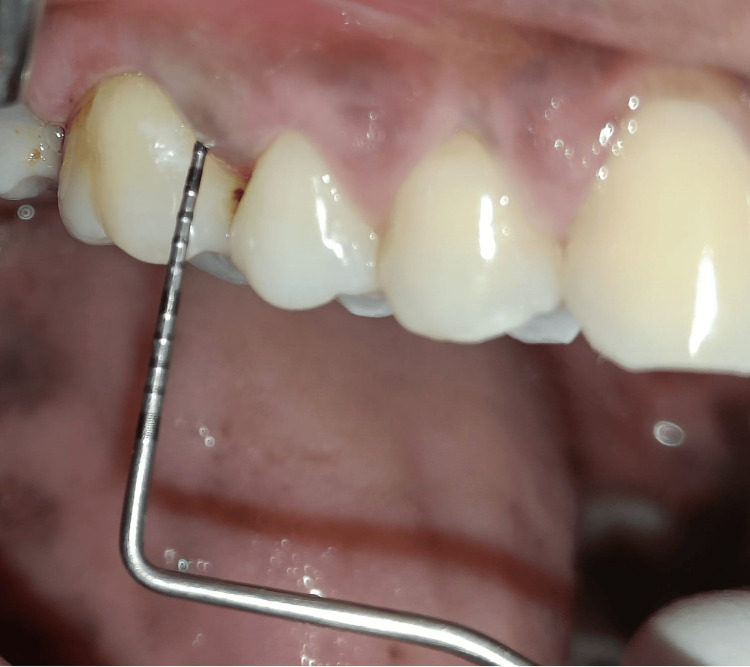
Postoperative periodontal examination

Clinical parameters including gingival index (Loe and Silness, 1963), plaque index (Loe and Silness, 1964) [11], periodontal probing depth, and healing index [12] were evaluated at baseline and after four weeks in both the groups. After baseline evaluation (Figure 1), all the patients underwent scaling and root planing. All the participants were instructed to follow their regular oral hygiene routines in addition to the prescribed regimen of mouthrinse according to the group they were assigned to. Participants were monitored every day as they were reminded to use the prescribed mouthwash each day via a recall message or call. The subjects were instructed to rinse their mouth with 10 mL of the mouthwash twice daily for one minute. The subjects were instructed to refrain from all other oral hygiene measures, including the usage of dental floss and chewing gum during the study period. Additionally, they were instructed to refrain from eating or drinking for a minimum of 30 minutes following mouth rinses. The examiner used a reminder sheet to help with the final recall of the participants to determine whether the patient was complying with the test. To determine how much mouthwash was used by the participants, they were recalled with the respective bottles containing mouth rinse assigned. All the clinical parameters were re-assessed after four weeks in both groups (Figure 2).

Statistical analysis

The level of significance for the statistical analysis was set at p<0.05. Inferential statistics was performed using the paired t-test and the software IBM SPSS Statistics for Windows, Version 27.0 (IBM Corp., Armonk, USA).

## Results

Intergroup comparisons between groups A and B at baseline and four weeks are depicted in Table 1 and Table 2, respectively. At baseline, intergroup comparisons showed no statistical significance. When the periodontal parameters were compared after four weeks following treatment, a significant difference between the groups was observed.

**Table 1:** Comparison of clinical parameters between groups A and B at baseline p<0.05: statistically significant; SD: standard deviation

Parameters	Group	Mean	SD	p-value
Plaque Index	Group A	2.21	0.26	0.07
	Group B	2.34	0.12	
Gingival Index	Group A	2.19	0.28	0.05
	Group B	2.35	0.13	
Probing Pocket Depth (mm)	Group A	4.87	0.61	0.35
	Group B	5.05	0.34	
Healing Index	Group A	2.72	0.38	0.58
	Group B	2.66	0.22	

**Table 2:** Comparison of clinical parameters between groups A and B after four weeks p<0.05: statistically significant; SD: standard deviation

Parameters	Group	Mean	SD	p-value
Plaque Index	Group A	1.03	0.27	0.00
	Group B	1.42	0.09	
Gingival Index	Group A	1.08	0.13	0.00
	Group B	1.43	0.08	
Probing Pocket Depth (mm)	Group A	3.76	0.62	0.00
	Group B	4.44	0.63	
Healing Index	Group A	4.75	0.24	0.00
	Group B	3.83	0.39	

Intragroup comparison from baseline to four weeks was performed using the paired t-test for groups A and B as depicted in Tables 3 and Table 4, respectively. Both Group A and Group B showed a significant reduction in all the clinical parameters after four weeks. Both groups showed an improvement in healing index scores after four weeks (p=0.00).

**Table 3:** Comparison of clinical parameters within Group A from baseline to four weeks p<0.05: statistically significant; SD: standard deviation; CI: confidence interval of the difference

Parameters	Paired Differences				p-value (2-tailed)
	Mean Difference	SD	95% CI		
			Lower	Upper	
Plaque Index	1.17	0.19	1.06	1.28	0.00
Gingival Index	1.10	0.15	1.01	1.19	0.00
Probing Pocket Depth (mm)	1.11	0.15	1.02	1.20	0.00
Healing Index	-2.02	0.32	-2.21	-1.83	0.00

**Table 4:** Comparison of clinical parameters within Group B from baseline to four weeks p<0.05: statistically significant; SD: standard deviation; CI: confidence interval of the difference

Parameters	Paired Differences				p-value
	Mean Difference	SD	95% CI		
			Lower	Upper	
Plaque Index	0.92	0.17	0.82	1.01	0.00
Gingival Index	0.92	0.17	0.82	1.01	0.00
Probing Pocket Depth (mm)	0.60	0.74	0.21	1.00	0.00
Healing Index	-1.17	0.32	-1.34	-1.00	0.00

Minimal to no side effects were seen in participants belonging to both groups. Some patients who belonged to Group B (CHX group) complained about altered taste sensations and reduced salivary flow. A patient with restorative material complained of staining and discoloration.

## Discussion

The current study was designed to assess the effectiveness of a folic acid-containing mouthrinse in periodontal disease. CHX is a widely used agent for plaque control due to its ability to disrupt bacterial cell membranes, causing leakage of cellular contents and preventing bacterial adsorption onto teeth [13,14]. This action mechanism is well-documented and supported by systematic reviews from Solderer et al. [15] and Pandiyan et al. [16], which stated that CHX significantly reduces biofilm formation in periodontitis patients.

Folic acid plays a critical role in cellular processes, particularly in the growth and proliferation of cells, which is vital for the repair and regeneration of periodontal tissues [17]. George et al. [18] have highlighted folic acid's importance in increasing the rate of epithelial turnover, contributing to faster healing and regeneration of the gingival epithelium. Moreover, previous studies [19] have shown that folic acid supplementation can enhance host resilience against gingival inflammation and ulceration, indicating its potential therapeutic benefits in periodontal disease management. However, while Khan et al. [20] explored the use of folic acid in a topical oral gel form, the potential of folic acid mouthwash in treating periodontal disease remains inadequately explored. Thus, this study sought to evaluate the efficacy of a folic acid-containing mouthrinse as a supplement to nonsurgical periodontal therapy.

The study population was divided into two groups: Group A used the folic acid-containing mouth rinse, while Group B used CHX. The mean ages of participants in groups A and B were 35.43±6.68 and 32.88±10, respectively, with no significant difference between the groups (p=0.41). This lack of significant age difference ensures that age-related variability does not confound the study results. At baseline (Table 1), all clinical parameters (such as plaque index, gingival index, and probing pocket depth) were comparable between the two groups, ensuring a fair starting point for both interventions.

After four weeks of treatment (Table 2), both groups exhibited significant reductions in plaque index and gingival index. Group B (Table 4) showed significant reductions in plaque index and gingival index after nonsurgical periodontal therapy, which was in accordance with the study results obtained by Bhat et al. [13]. Participants in Group A displayed a significant decrease in clinical parameters such as plaque index, gingival index, and probing pocket depth (Table 3). These findings corroborated the results obtained by Keceli et al. [10], although they evaluated the effect of systemic folic acid supplementation on periodontal disease, whereas the current study evaluated the efficacy of topical folic acid application in periodontitis patients, which has not been extensively explored. Both groups showed significant improvements in healing index scores after nonsurgical therapy. These improvements suggest that both mouthrinses positively affect periodontal healing and reduce periodontal pocket depth, a critical measure in managing periodontitis. Only a few studies have employed the healing index to evaluate tissue response to nonsurgical periodontal therapy, especially with adjunctive mouthrinses. Penmetsa et al. [12] in their study evaluated the healing index before and after nonsurgical therapy under different magnifications and showed a reduction in clinical parameters after periodontal treatment.

The limited existing literature on the efficacy of folic acid as a local agent in periodontitis makes it difficult to directly compare our findings with other studies. Nonetheless, the significant improvements observed in the folic acid group suggest that a folic acid-containing mouthrinse could be a valuable adjunct to standard nonsurgical periodontal therapy. However, a major drawback of the study is the use of a commercially available mouthwash containing folic acid along with other active ingredients, which may have also contributed to the observed results in terms of plaque control. To determine the genuine effectiveness of folic acid in periodontal therapy, future studies should focus on mouthwashes containing only folic acid. Further research is warranted to confirm these preliminary findings. Future studies should include larger sample sizes, longer follow-up periods, and potentially a broader range of clinical parameters to fully elucidate the benefits of folic acid mouthrinse in periodontal therapy. Additionally, exploring the molecular mechanisms underlying the effects of folic acid on periodontal tissues could provide deeper insights into its therapeutic potential and optimize its use in clinical practice.

## Conclusions

Comprehending the etiology and therapeutic approaches of chronic diseases such as periodontitis may facilitate improved therapeutic outcomes. Given the effectiveness of evidence-based clinical practice and the growing availability of alternative therapies, our approach to holistic dental practice, research, and training has to be redefined with the use of newer alternative therapies. In contrast to the conventional chemical plaque control agents like CHX, mouthwashes containing supplements like folic acid and antioxidants may also be effective in periodontitis. Hence, newer agents that can overcome the infirmity of using CHX should be further explored and evaluated for their effect in periodontal disease control.
